# A Platform for the Remote Conduct of Gene-Environment Interaction Studies

**DOI:** 10.1371/journal.pone.0054331

**Published:** 2013-01-18

**Authors:** John Gallacher, Rory Collins, Paul Elliott, Stephen Palmer, Paul Burton, Clive Mitchell, Gareth John, Ronan Lyons

**Affiliations:** 1 Institute of Primary Care and Public Health, Cardiff University, Cardiff, United Kingdom; 2 Clinical Trials Service Unit, Oxford University, Oxford, United Kingdom; 3 Department of Epidemiology and Biostatistics, Imperial College, London, United Kingdom; 4 Department of Health Sciences, Leicester University, Leicester, United Kingdom; 5 NHS Wales Information Service, Cardiff, United Kingdom; 6 Institute of Life Science, Swansea University, Swansea, United Kingdom; Vanderbilt University, United States of America

## Abstract

**Background:**

Gene-environment interaction studies offer the prospect of robust causal inference through both gene identification and instrumental variable approaches. As such they are a major and much needed development. However, conducting these studies using traditional methods, which require direct participant contact, is resource intensive. The ability to conduct gene-environment interaction studies remotely would reduce costs and increase capacity.

**Aim:**

To develop a platform for the remote conduct of gene-environment interaction studies.

**Methods:**

A random sample of 15,000 men and women aged 50+ years and living in Cardiff, South Wales, of whom 6,012 were estimated to have internet connectivity, were mailed inviting them to visit a web-site to join a study of successful ageing. Online consent was obtained for questionnaire completion, cognitive testing, re-contact, record linkage and genotyping. Cognitive testing was conducted using the Cardiff Cognitive Battery. Bio-sampling was randomised to blood spot, buccal cell or no request.

**Results:**

A heterogeneous sample of 663 (4.5% of mailed sample and 11% of internet connected sample) men and women (47% female) aged 50–87 years (median = 61 yrs) from diverse backgrounds (representing the full range of deprivation scores) was recruited. Bio-samples were donated by 70% of those agreeing to do so. Self report questionnaires and cognitive tests showed comparable distributions to those collected using face-to-face methods. Record linkage was achieved for 99.9% of participants.

**Conclusion:**

This study has demonstrated that remote methods are suitable for the conduct of gene-environment interaction studies. Up-scaling these methods provides the opportunity to increase capacity for large-scale gene-environment interaction studies.

## Introduction

Genetic epidemiology is a dynamic science with a fast changing knowledge base and technology base which has moved beyond the genome to the investigation of gene-environment interactions (GxE). [1] GxE studies offer the prospect of robust causal inference through both gene identification and instrumental variable approaches. [2] As such they are a major and much needed development.

For complex (non-Mendelian) disease, GxE studies are dependent on the recruitment of large numbers of individuals in the pursuit of small effect sizes. They also require new data collections with increasingly diverse and detailed phenotyping. With increasing awareness of the genetic and epigenetic complexity underlying disease, the importance of GxE studies grows. Conventional epidemiologic methodology, involving direct (face-to-face) contact with participants, is highly resource intensive which limits the opportunity for new GxE studies. For the full benefits of GxE studies to be realised, therefore, new methods are required. Although large infrastructures have been proposed as one means of increasing cost-effectiveness, [3] alternative and more radical approaches may also be considered.

The online environment presents an, as yet, underexploited opportunity to conduct GxE studies remotely, that is, without direct participant contact, offering the potential to significantly reduce costs. The online environment is also flexible and can be rapidly responsive to emerging hypotheses. For reasons of privacy and convenience the online environment may be preferable to participants.

Moreover, for assessments of episodic outcomes, the online environment may be a more sensitive medium than a clinic assessment. Current technological limitations would preclude the testing of some hypotheses, particularly those involving the specialist preparation of bio-samples or direct measurement of performance. However, a growing number of hypotheses could be rigorously tested entirely remotely. These would include genetic and epigenetic hypotheses. Cognitive performance related hypotheses are particularly suitable for testing remotely.

The prospect of conducting epidemiologic studies remotely was first mooted by Rothman who proposed the internet as a suitable vehicle for this purpose, [4] but only recently has internet-based methodology been systematically developed. [5], [6] Although internet-based epidemiologic studies are being conducted, [7] remote GxE studies, which require bio-sampling, consent for genotyping and long-term follow-up are not. Our previous work has shown that conducting GxE studies remotely is acceptable to the public [8], [9].

In this paper we report the field test of a platform designed to conduct GxE studies entirely remotely. The platform is an adaptation of the methods and thinking underlying UK Biobank which provided a benchmark for recruitment, consent and follow-up procedures which would be suitable for large scale application [10].

## Materials and Methods

### Ethics Statement

This study received ethical approval from the South East Wales Research Ethics Committee.

### Sample

A random sample of 15,000 men and women aged 50+ years and living in Cardiff UK, was selected from the National Health Service Administrative Register. Of these, Welsh Assembly Government figures, derived from the 2007 Living in Wales Survey of households, suggest that 6,011 (40%) were connected domestically to the internet at the time of the study [11].

### Recruitment

Participants were mailed an invitation letter and participant information leaflet on a single occasion only. The invitation was to participate in the ‘Age Well, Feel Good’ study of successful ageing. The mailing was conducted by the NHS on behalf of the research team to preserve the anonymity of participants. The identity of participants only became known to the research team when given by participants upon completion of the on-line consent procedure. Each invitation letter included a link to the study web-site. Embedded within the link was a universal resource locator (URL) used as a participant specific identifier. This allowed the browsing behaviour of individual participants to be analysed.

### Participant Support

To provide participant support within a remote study paradigm, a free-phone helpline was made available. Calls were received by the Cardiff University Participant Resource Centre (PRC), a science dedicated call and mailing centre based at Cardiff University. Calls were handled by specially trained operators using scripts prepared by the research team. Escalation procedures allowed participants to speak to the study’s principal investigator if required. The PRC was operated to commercial standards with all calls being recorded for the purposes of quality control and quality assurance, and dealing with complaints.

### Website

The website had four design constraints. First, for reasons of security, it had to be entirely under the control of the research team. For this reason all code was written by the research team (CM). Second, it had to be flexible and adaptable for use in a wide range of epidemiological studies. To achieve this, a modular architecture was used enabling the easy change of content. For this particular study the assessment was organised into 8 themes comprising a total of 22 measurement modules. The themes covered demographics, health, cognitive performance, psychological state, social support, leisure time activity, diet and the built environment. Third it had to be functional on a wide range of web-browsers and computer platforms as well as on dial-up and broadband connections. To achieve this we avoided the use of video, Flash, and commercial web-authoring tools. We also adhered to current web standards (HTML4, XML1.0). Fourth, it had to engage the target population as inclusively as possible, in this case middle-aged and older people with either English or Welsh as their first language. It was assumed that the target population would be computer literate but not computer sophisticated and may have slight visual impairment. For these reasons a simple colour palette and large font-size were used and all pages were designed to fit onto the screen without the need for scrolling. The site was multi-lingual.

### Consent

The link within the invitation letter took participants to the study homepage with links to background information to the study and frequently asked questions. The background information process led to an opportunity to consent to participate in the study. The background information included a brief description of the study requirements, confidentiality and withdrawal procedures. Consent was requested for asking questions, following health related records, re-contact and donating a bio-sample for genetic and biochemical analysis. Once consent had been given, the participant’s name and contact details were requested and once these were given a study membership number was issued.

### Assessment

The website was organised into 8 themes which could be accessed in any order. These themes were designed to have face-validity to participants. Within each theme there was a sequence of modules, each covering a more specific area of measurement. Participants did not have to complete all 22 modules in a single session and could use their study membership number to return to the site repeatedly, however, each module had to be completed at a single session. The content was designed to cover a range of variables generally of interest to the epidemiology of ageing. Items were also included on health service delivery evaluation to investigate the suitability of this medium for fungible studies. [12] A range of presentation formats was used to assess the general utility of a web-based approach. The Cardiff Cognitive Battery, which has been designed specifically for epidemiologic use, was used for cognitive testing. Deprivation was assessed using the Welsh Index of Multiple Deprivation which combines indicators of income, employment, health, education, housing, the physical environment and access to services at the level of the lower super output area (LSOA); a UK population census tract describing 1500 inhabitants. Cardiff has 202 LSOAs.

### Bio-sampling

The impact of remote bio-sampling for genetic determination on participation in a remote study is unknown. For this reason, nested within this study was a randomised trial comparing participation between requesting a dry-blood sample, requesting a buccal cell sample, and not requesting a bio-sample. Due to participant identities not being known to the research team until the study was joined, randomisation was conducted at the point of first contact with 5,000 participants being allocated to each arm of the trial. For participants who were invited to donate a bio-sample, the appropriate bio-sampling kit was mailed to them once they had joined the study. Due to supply difficulties, bio-sampling kits were not mailed to participants until the fourth month of the study.

### Follow-up

Follow-up was by establishing linkage with the National Health Service Administrative Register. Follow-up through re-contact has not yet been attempted.

### Statistical Methods

Age was recorded in years and grouped into 10 year bands. The Welsh Index of Multiple Deprivation score for participants for the LSOA of their address as a quintile scores based on the ranking of all LSOAs in Wales, with 1 describing least deprived LSOAs and 5 describing most deprived LSOAs. Comparison of means between clinic and web administered cognitive tests and wellbeing scores were made by t-test. Differences between means were detected at p<0.05.

## Results

### Participant Support

The PRC received 200 calls covering a range of topics. Most were requests for further details of the study and confirming the study’s bona-fides. There were, however, 7 (0.05%) complaints at being invited to participate.

### Consent

The use of individual URLs enabled the passage through the consent procedure to be analysed. Altogether 92 persons visited the site and did not join the study. Drop-outs occurred at pages giving the study overview (35%), at the point of consent (37%) and at the point of giving personal details (11%). All other drop-outs were evenly spread between pages describing bio-sampling, record linkage, withdrawal or re-contact (17%). However, not all participants joined the study using their personal URL. This may have been for a variety of reasons, including firewall issues or using search engines to check out the study on the web prior to joining, and then joining directly from the homepage. It may also be that some participants had heard about the study virally rather than by invitation letter.

### Response

**Figure 1:**
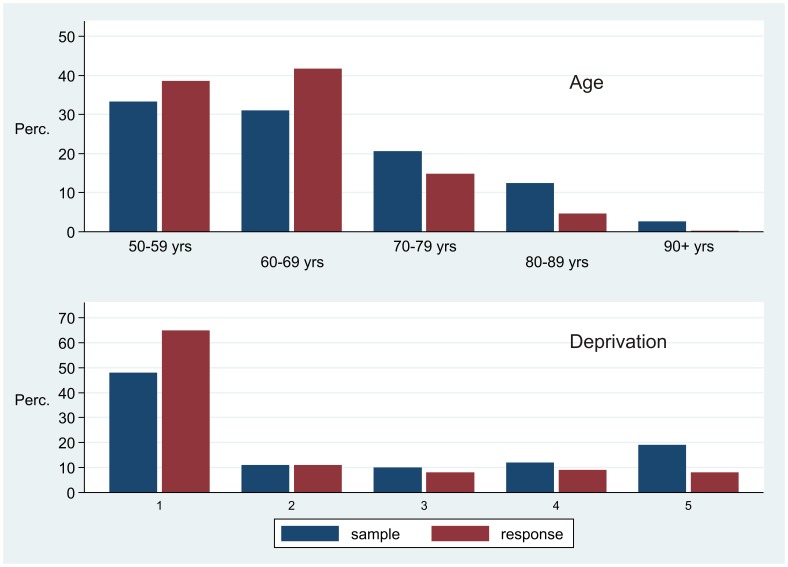
Distribution of age and deprivation according to invitation and response.

After 22 weeks 663 participants had joined the study. Sampling bias was evaluated in terms of age, sex and social deprivation (table 1). The mailed sample was 51.7% female whereas the study sample was 49.8% female (χ^2^(1) = 0.96; p = 0.32). The age distribution of the study sample was over-represented at the younger end (χ^2^(4) = 98; p<0.001), although several of the study sample were in their 80′s and 90′s (figure 1). The study sample was also under-represented in high deprivation areas (χ^2^(4) = 108; p<0.001), but the entire range of deprivation was found (figure 1).

**Table 1:** Evaluation of sampling bias using remote recruitment.

Variable		Response		P value(2 sided)
		invited	consented	
Age (years)	50–59	5,251 (35%)	267 (40%)	<0.001
	60–69	4,524 (30%)	280 (42%)	
	70–79	3,020 (20%)	85 (13%)	
	80–89	1,847 (12%)	29 (4%)	
	90+	358 (3%)	2 (0.5%)	
Gender	female	7,758 (52%)	330 (49.8%)	0.33
	male	7,242 (48%)	333 (50.2%)	
Deprivation*	1 (least deprived)	7,156 (48%)	441 (67%)	<0.001
	2	1,646 (11%)	75 (11%)	
	3	1,514 (10%)	43 (7%)	
	4	1,886 (12%)	56 (8%)	
	5 (most deprived)	2,798 (19%)	48 (7%)	

* Welsh Index of Multiple Deprivation.

**Figure 2:**
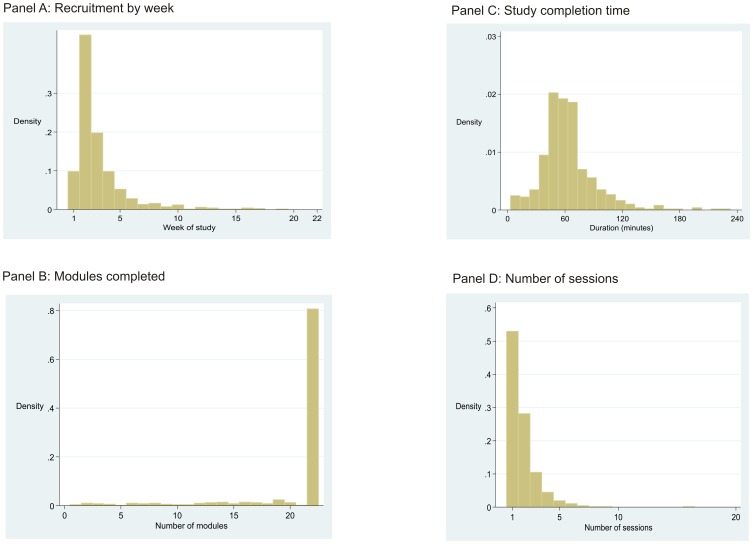
Participation process indicators.

Of the 663 participants, 576 (87%) joined within 4 weeks of the mailing and 636 (96%) within 8 weeks of the mailing (figure 2, panel A). The 663 participants represent 4.5% of the mailed sample and 11.0% of those estimated to be internet connected. The individual URL was used by 549 participants with 118 not using the URL and going directly to the study homepage.

### Assessment

**Figure 3:**
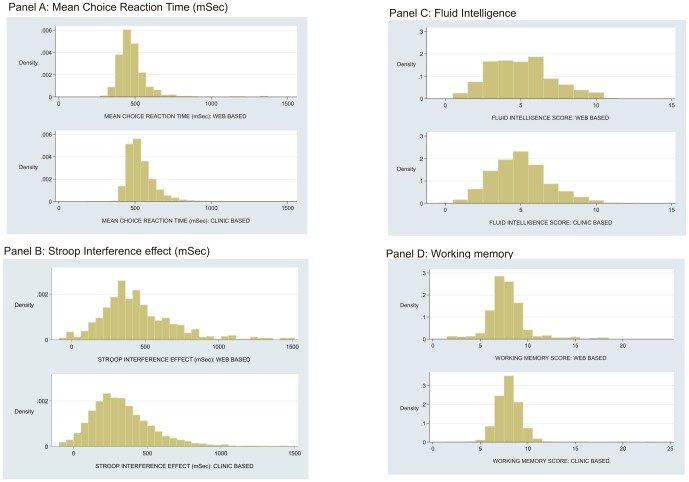
Distribution of cognitive performance according to web or clinic administration.

Of the 663 participants who joined the study, 642 provided data. For the 642 who provided data completion rates varied between modules from 99% for demographic details to around 85% for health service evaluation (table 2). Although all questions had a ‘prefer not to answer’ option, this was rarely used and the pattern of missingness was largely monotone within modules. The completion rates were uniformly high for a variety of formats including evaluation of dental photographs (93%), observation of the built environment (86%) and cognitive testing (84%–90% according to test). All 22 modules were completed by 502 (75%) participants (figure 2, panel B). The mean completion time overall (regardless of number of modules completed) was 63.8 minutes (SD = 36 minutes) (figure 2, panel C). The distribution of completion times was positively skew with a 95% range between 22.8 and 116.7 minutes. The 95% range for the number of sessions was between 1 and 4 (figure 2, panel D). For several of the cognitive tests, comparison data were available for 9,234 men and women aged 18–65 years recruited to the Airwave study, which used clinic-based assessment. Web-based mean reaction time was slightly faster (50 mSec, p<0.001), this was likely due to the use of a tablet PC in the Airwave study. The stroop interference effect was slightly stronger (120 mSEc, p<0.001) (table 3). There was no difference for fluid intelligence or working memory. Of greater interest, given the difference in population samples between cohorts, is that the distributions for all cognitive tests were closely similar between testing environments (figure 3). Rasch analysis of the fluid intelligence scale found that the order of item difficulty was identical between administration formats (not shown).

**Table 2:** Module completion in 642 participants who provided data.

Themes	Modules (items)	Variables	Completion rates*
Your circumstances	Demographics (16)	Marital status, etc.	97–99%
	Sleep (7)	Sleeping habits	95%
Health	General health questions (35)	Perceived health, disability, ADL	98%
	Major health questions (45)	Doctor diagnosed illness	94%
	Sight and hearing (9)	Difficulties affecting lifestyle	93%
	Dental (15)	Photo identification of dental illness	90–93%
Thinking	Mood (14)	HADS**	95%
	Fluid intelligence (12)	Numeric and verbal reasoning	86%
	Reaction time (60)	Two choice	90%
	Episodic memory (12)	Paired associates learning	86%
	Working memory (1–12)	Forward digit recall	87%
	Attention (30)	Stroop non interference reaction time	84%
	Attention (30)	Stroop interference reaction time	84%
Feelings	Wellbeing (22)	Life satisfaction, Self esteem, Self efficacy	90–92%
People	Social support (19)	Emotional and practical support	91–94%
Leisure time	Leisure activity (33)	Physical and sedentary activity	93%
	Smoking (16)	Current and past smoking behaviour	91%
	Diet (18)	Food frequency questionnaire	93%
	Alcohol (12)	Frequency and type of consumption	91%
Place	Perceived built environment (19)	General neighbourhood quality	88%
	Observed built environment (24)	Observed street quality from front door	86%
Health and social care	Service evaluation (11)	GP, hospital, pharmacy and dental	82–86%

* Range of completion given when completion varied within module according to item.

** Hospital Anxiety Depression Scale.

**Table 3:** Comparison of scores according to web-based or clinic-based administration.

		Web based		Clinic based				P
Domain	Variable	Age Well Feel Good Study		Airwave Study)		Caerphilly Study)		For difference between mean values (2 sided)
		(n = 540*)		(n = 9,234)		(n = 964)		
		Mean (SD)	Cronbach’s α	Mean (SD)	Cronbach’s α	Mean (SD)	Cronbach’s α	
CognitiveFunction	Fluid intelligence score	5.03 (2.08)	–	4.96 (1.85)	–	–	–	0.4
	Working memory score	7.79 (2.09)	–	7.96 (1.26)	–	–	–	0.07
	Two choice reaction time (mSec)	482 (106)	–	538 (107)	–	–	–	<0.001
	Stroop Interference effect (mSec)	460 (356)	–	336 (703)	–	–	–	<0.001
Wellbeing	Life satisfaction score	25.9 (6.2)	0.89	–	–	26.3 (6.3)	0.87	0.2
	Self Efficacy score	26.5 (4.6)	0.87	–	–	25.1 (5.0)	0.85	<0.001
	Self esteem score	45.2 (8.1)	0.85	–	–	44.7 (8.7)	0.92	0.08

* The sample size varies according to analysis between 540 and 594.

**Figure 4:**
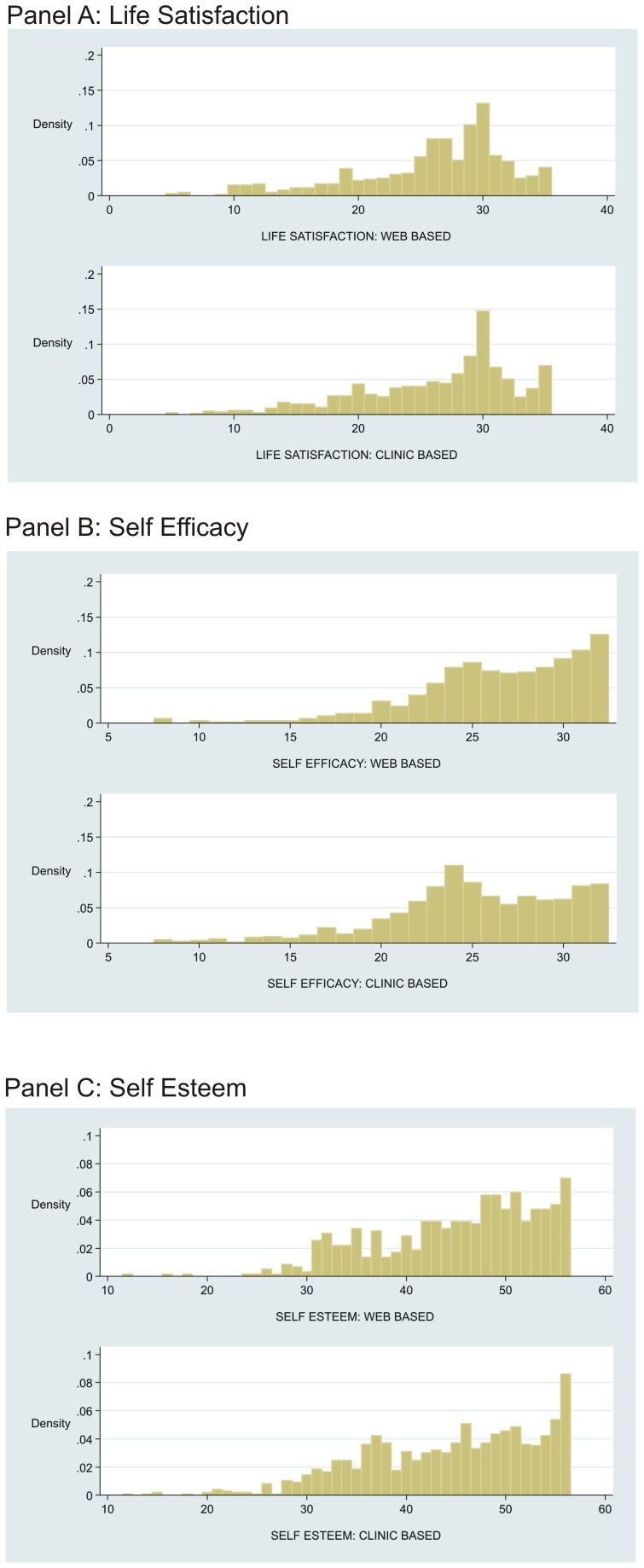
Distribution of well-being scores according to web or clinic administration.

Comparison wellbeing data were available for 964 men aged 65–80 years from the Caerphilly Prospective Study which also used clinic-based assessment. Web-based mean self-efficacy score was slightly higher (1.5 points, p<0.001) (table 3). There were no differences for self-esteem or life-satisfaction between testing environments. Inter-item reliability was high for all the wellbeing scales (α≥0.85) and highly comparable between web and clinic based methods (table 3). Scale distributions were closely similar between formats (figure 4).

### Bio-sampling

The trial of the impact of making a bio-sample request on recruitment cannot be analysed strictly on an intention-to-treat basis as, necessarily, randomisation occurred at invitation rather than post-consent. Of the 549 participants for whom randomisation status was known, 196 (36%) were respondents who were not asked to provide a bio-sample; 182 (33%) were respondents who were asked to donate a buccal cell sample of whom 136 (75%) did so; whilst 171 (30%) were respondents who were asked to donate a dry blood sample, of whom 119 (70%) did so.

### Follow-up

Linkage was achieved for 662/663 (99.9%) of participants.

## Discussion

The utility of a platform for the remote conduct of GxE studies has been demonstrated. In a representative sample of older people, 11% of those estimated to be connected to the internet consented to participate of whom 99.9% provided data. A randomised trial nested within the study found that the request of a bio-sample had little effect on participation. The donation rate of those who agreed to provide a bio-sample was over 70%.

### Governance

There were several challenges to overcome before ethical approval for this study was obtained. These were largely due to the combination of technologies that were being proposed. The major issue was the linking of genetic information with clinical records. The commitment to use a fully secure and de-identified database for linkage and subsequent analyses was considered to provide adequate protection for participants. [13] A further issue was the possibility of identity fraud. However, it was accepted that the likelihood of this was low and, due to the use of de-identified data for analyses, the consequence would be to add noise to the data rather than pose any risk to the individual. On this basis obtaining consent without a ‘wet’ signature was also approved. A supporting argument for conducting the study remotely was the availability of telephone support which enabled any prospective participant to discuss the study with the research team, including the Principal Investigator. This facility is not usually available in large (usually multi-centre) studies using face-to-face methods.

### Acceptability

The extremely low rate of complaint (0.05%) strongly confirmed the evidence of previously conducted qualitative studies that, in principle, remote methods are acceptable to the public. [8], [9] Complaints were almost entirely due to a mis-perception that the research team had access to personally identifying information prior to it being given by participants. As initial contact was achieved via a third party, this issue was easily addressed. Although the complaint rate may not fully reflect the acceptability of the study, it reflects a reassuringly high level of acceptability. The high completion rates for all modules (82%–99%), which differed widely in format and content suggests that a web platform has application to a wide range of epidemiologic studies.

### Sampling Bias

The response rates achieved here are difficult to assess accurately as it was not known beforehand which invitees had internet access. Based on Government figures it was likely that 6,011 invitees were internet connected giving a response rate of 11%. The Government figures were based on a representative sample of 7,728 households throughout Wales (reflecting a 71% response rate) surveyed in 2007. In our study, in terms of the mailed sample 4.5% responded. Given that this is an older population with limited internet connectivity, this response may be considered comparable to the 5.4% achieved in UK Biobank. [10] Overtime differences in selection bias between web-based and face-to face samples [14] are likely to reduce as a greater proportion of the population becomes connected. [15].

Remote methods, in which recruitment costs are minimised and participation restricted by computer access, bring the issue of selection bias into sharp focus. A helpful distinction is between descriptive and etiologic studies. The former describe specific populations. For descriptive studies to achieve unbiased estimates of prevalence, incidence or normal ranges, representative samples are required. Etiologic studies investigate mechanisms that occur across populations. For these studies heterogeneous population samples are required so that the range of values for an exposure is available to the analysis. Also required is the non-differential ascertainment of incident outcomes. GxE studies are not designed to describe specific populations. As such, response rates affect cost rather than bias. Similarly, remote methods are not generally suitable for descriptive studies but for testing etiologic hypotheses. Our study has demonstrated, in terms of age, sex and deprivation, that heterogeneity can be achieved using remote methods.

Heterogeneity in this study was achieved by dint of numbers rather than by a systematic method, such as random sampling. It is unlikely that the heterogeneity available to the analysis would have been materially affected had we used a different recruitment method, such as a media campaign, provided we recruited sufficient numbers.

Rather than requiring all studies to be representative, a preferred strategy is to identify mechanisms using etiologic studies and then apply that knowledge to specific populations. Clarifying and separating these goals enables more efficient study design, as etiologic studies may be conducted without the unnecessary burden (and cost) of having to achieve representativeness, and descriptive studies may be conducted without the unnecessary burden (and cost) of having to achieve large sample size. By separating these goals each design can be prosecuted more efficiently.

### Information Bias

A further issue is the validity and reliability of web-based assessment. Evidence largely supports comparability between measurement media for questionnaires. [16], [17] We found very little difference between the distributions of several self evaluation questionnaires between web-based and clinic-based methods. Less is known in relation to cognitive testing. In part, this is due to the difficulties of cognitive testing in both face-to-face and remote contexts and the wide range of cognitive tests in use. In this study a cognitive battery, designed specifically for epidemiologic use, was compared between web-based and clinic-based assessment. The distributions were closely similar between measurement contexts. Although these between cohort comparisons are indirect (neither randomised nor repeat measurement) they are the best comparisons available.

### Bio-sampling

Requesting a bio-sample appeared to have only a small effect on participation. It appears that if the rationale for the study is persuasive, the donation of genetic material is not problematic. Furthermore, although the donation of dried blood was not a painless exercise, this also was widely acceptable. The actual donation rates (70–75% according to sample), although useful for planning purposes, are likely to be conservative due to the passage of several months between most participants joining the study and being mailed the sampling kit.

### Follow-up

Etiologic studies also require non-differential ascertainment of outcomes. In practice, this means very high follow-up rates. Here the principal follow-up method was by record linkage. The high level of linkage achieved may not be surprising given the initial invitations were based on the National Health Service Administrative Register database. However, although follow-up by electronic linkage may virtually eliminate attrition, for many hypotheses e.g. those involving inadequate routine measurement of outcomes such as common mental disorder, or those involving change over time such as cognitive decline, follow-up by re-contact is required.

### Cost-effectiveness

This study cost around £100 per participant. This mostly involved IT development costs, reflecting the cost structure of remote studies being front-loaded compared to traditional methods. For larger studies, on the basis of subsequent cost being due largely to mailing and bio-sampling, we crudely estimate, on the basis of a 10% response rate, and a 70% bio-sample donation rate, that recruitment and bio-sampling for a GxE study of 50,000 would cost around £15 per participant over an 18 month period. These *per capita* costs would be reduced if the response and donation rates were improved or if the study size was increased. Costs would likely be reduced further if recruitment was achieved without an initial mailing i.e. through a media campaign, although this is conjectural. By comparison, costs using traditional methods would be additional to those estimated here. Currently, initial contact costs would be similar, as response rates between this study and UK Biobank were comparable. However, with time, as internet connectivity becomes more prevalent, response rates from mailed invitations for internet studies are likely to increase, thus reducing contact costs. Additional costs for traditionally conducted studies would include premises hire (for assessment and bio-sampling), staff (clinicians for bio-sampling as well as technicians assessment and for sample preparation). In relation to bio-sampling, depending upon the sample, remote methods will usually generate lower costs. A mailed dry-blood sample, for example, requires less processing and storage space than a wet sample which requires venepuncture and processing consumables, transport to a repository, and long term very low temperature storage. Although we are not in a position to put numbers to these cost headings, the additional costs are substantial. Finally, linkage follow-up costs will be closely similar between remote and traditional methods, depending upon the quality of personally identifying information available.

### Limitations

Many limitations remain to be overcome before remote methods are as clearly understood and accepted as face-to-face methods. Although we have achieved linkage we have not downloaded data, but the system proposed for this is currently being used in a large e-cohort of births in Wales. [18] We have not tested our ability to re-measure participants. The incentivisation of participants for re-measurement is a critical issue for web-based cohorts. Options available include the provision of feedback at the individual, as well as study, level. A further limitation is the quality of available bio-sample. We have shown that donation of either buccal cell or dry blood is feasible. In addition, other pilot work (not shown) has demonstrated that the remote donation of saliva for genetic determination is also feasible. Any of these methods is adequate for the retrieval of DNA allowing genotyping and the use of genotypes as instrumental variable in Mendelian randomisation studies. However, dry blood may also be used for an increasingly broad range of assays. Although dry-blood may not currently be suitable for cutting-edge molecular biology, it is suitable for assessing many established risk factors and so is informative in GxE studies. A particular limitation of remote studies is objective measurement. Although this has been largely solved for cognitive performance, remote methods and devices for assessing anthropometry, physical activity and other aetiologically important risk factors need to be developed before remote methods will have a broad based epidemiologic impact.

### The Way Forward

GxE studies offer the prospect of robust causal inference through both gene identification and instrumental variable approaches. [19] As such they are a major and much needed epidemiologic development. The value of remote methods is increasingly recognised and they are being adopted in a variety of epidemiologic contexts. [20]–[23] We have shown, that even in their infancy, the application of remote methods can be extended to GxE studies as a cost-effective alternative to traditional approaches. In acknowledging the need for further methodological refinement, we expect this greater efficiency to improve as the field matures. We have also shown evidence that over a range of psychological and cognitive assessments the data are comparable with those collected face-to-face. We expect the range of remotely assessed measures to increase, particularly with the development of small objective measurement devices such as accelerometers, with remote measures being preferable in many areas. By these means, even in an age of fiscal restraint, remote methods provide an opportunity to increase the capacity for GxE studies; offering the prospect of GxE studies going beyond broad-based investigations of chronic disease to more finely niched investigations focussing on more refined outcomes in more closely defined population strata. [24] In an age of increasingly diverse public expectation, a growing desire for robust inference, and ubiquitous information technology, a bourgeoning of remotely conducted GxE studies is not an unrealistic expectation.
